# Fuel Cells: A Real Option for Unmanned Aerial Vehicles Propulsion

**DOI:** 10.1155/2014/497642

**Published:** 2014-01-30

**Authors:** Óscar González-Espasandín, Teresa J. Leo, Emilio Navarro-Arévalo

**Affiliations:** ^1^Instituto Nacional de Técnica Aeroespacial “Esteban Terradas” (INTA), Carretera Ajalvir km.4.,Torrejón de Ardoz, 28850 Madrid, Spain; ^2^Departamento de Sistemas Oceánicos y Navales, ETSI Navales, Universidad Politécnica de Madrid, Avenida Arco de la Victoria 4, 28040 Madrid, Spain; ^3^Departamento de Motopropulsión y Termofluidodinámica, ETSI Aeronáuticos, Universidad Politécnica de Madrid, Plaza Cardenal Cisneros 3, 28040 Madrid, Spain

## Abstract

The possibility of implementing fuel cell technology in Unmanned Aerial Vehicle (UAV) propulsion systems is considered. Potential advantages of the Proton Exchange Membrane or Polymer Electrolyte Membrane (PEMFC) and Direct Methanol Fuel Cells (DMFC), their fuels (hydrogen and methanol), and their storage systems are revised from technical and environmental standpoints. Some operating commercial applications are described. Main constraints for these kinds of fuel cells are analyzed in order to elucidate the viability of future developments. Since the low power density is the main problem of fuel cells, hybridization with electric batteries, necessary in most cases, is also explored.

## 1. Introduction

Unmanned Aerial Vehicles (UAVs), encouraged by recent technological developments, have seen a dramatic interest boost in recent years and are already considered as an integral and indispensable part of modern armed forces [1] with an increasing number of dual use and civil applications [2, 3]. Most developed countries have already acquired UAVs or plan to do so soon. Current propulsion systems are based on different types of internal combustion engines fed by fossil fuels, but with the global energy situation, preceded by the energy crises of the 70s and strategic incentives to make alternative propulsion systems, fuel cells have started to be introduced. These have advantages in terms of endurance, efficiency, emissions, and stealth which make them ideal for UAV applications [4]. Starting from the premise that important energy and environmental problems in our society exist, the advantages and disadvantages of fuel cells as an alternative for UAV propulsion systems are going to be analyzed.

**Figure 1:**
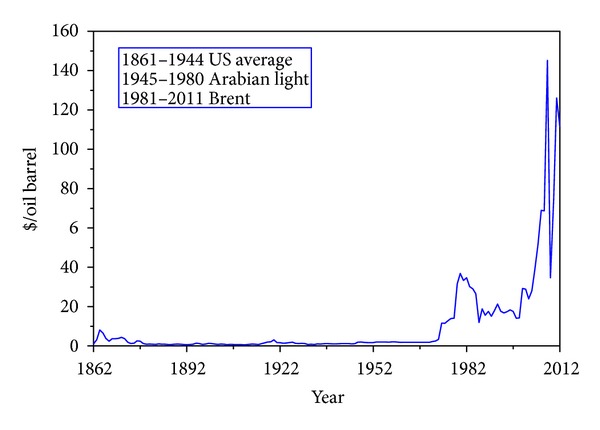
Historical world oil prices in the period 1861–2012.

Fossil fuels pose a serious environmental and economic problem. They are the main cause for the increase of CO_2_ presence in the atmosphere, declared to be one of the most important culprits of global warming and the atmospheric emissions of other pollutants [7]. The current economic and financial crisis has been aggravated by the high energy prices [8], represented in Figure 1. There is therefore an urgent search for new energy policies based on the diversification of energy sources and their origin, energy saving policies, and the use of efficient energy conversion systems.

Of course, the world of aviation is not an exception to the above considerations. UAVs are in their nascent stage of development (in fact, their regulations are in process of being written), although the implementation of fuel cell propulsion systems is more advanced than in conventional aircrafts. The main reason is the fact that UAVs are unmanned, thus, weighting comparatively less and not needing the life support systems for the crew and passengers, making them ideal for this new technology. Also, fuel cells are still too heavy to propel any large aircraft; they have a lower power density when compared with conventional turbines [4]. In a military setting, there are other key operational advantages such as stealth and a lower thermal signature.

## 2. UAV Propulsion Systems

### 2.1. Elements of the Propulsion System

The propulsion system of a UAV consists of the following elements:

- energy source: chemical fuels (fossil fuels, biofuels, and chemicals), electricity, solar energy (in conjunction with photovoltaic cells), hydrogen, methanol, and energy mechanics;
- storage media: tanks, batteries, capacitors, metal hydrides, and so forth;
- mechanical energy converter: internal combustion engine, and fuel cell + electric motor;
- lift/thrust converter: rotor, fan, propeller, jet engine, and so forth.

Lift/thrust conversion systems are closely linked to the type of aircraft (fixed wing, rotary, lighter than air, etc). In addition, propulsion systems usually include power control, rpm control, heat management system, and an auxiliary electrical power generator.

**Figure 2:**
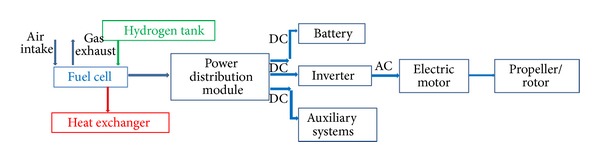
Diagram of the elements of a propulsion system with a mechanical energy converter based on a fuel cell + electric motor combination [5] (adapted from [6]).

An example is the propulsion system shown in Figure 2, where the hydrogen is the energy source. The storage medium is the hydrogen tank. The mechanical energy converter is based on the combination of a fuel cell + electric motor, and the converter to lift/thrust consists of a propeller. This is the typical architecture of a fuel cell based propulsion system used in UAVs.

### 2.2. Types of Propulsion System in UAVs

Despite the recent boom in greener propulsion systems (electric, solar, hybrids, hydrogen internal combustion engine, etc.), the vast majority of current UAV engines are still driven by conventional internal combustion engines, normally fed with fossil fuels [19]. As an alternative to conventional propulsion, three main types of propulsion systems can be considered in UAVs:

- alternative thermal systems: where different thermodynamic cycles, fuel, or engine types can be used (e.g., spark-ignition reciprocating engines fuelled by gasoline);
- electrical systems: where the power required is obtained through an electric motor and power is generated or stored in different ways;
- hybrid systems: combining any of the systems listed above, even the same type (e.g., a combination of fuel cell and battery or Regenerative Fuel Cell Systems, RFC, which combine fuel cell, battery, and photovoltaic cells).

### 2.3. Hybridization

**Figure 3:**
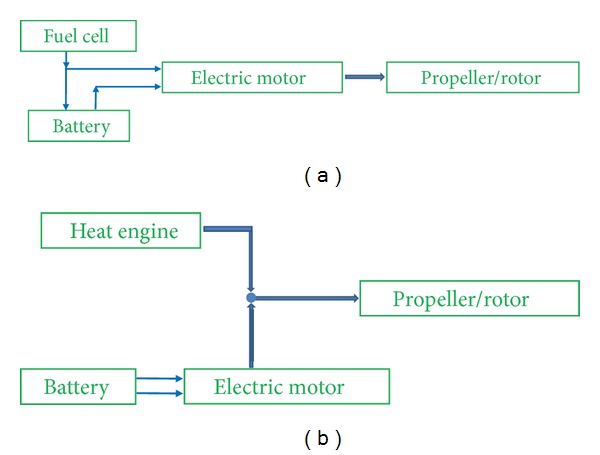
Diagram examples of serial (a) and parallel (b) hybrid propulsion systems [5] (adapted from [6]).

Hybridization can be achieved by combining a heat engine/electric motor with batteries [20] or fuel cell/electric motor with batteries. In either case, the need to install motors, batteries, inverters, control units, and so on makes the system heavier, bulkier, and more expensive than others. Nonetheless, it still has advantages, especially from the standpoint of a definitive adoption of alternative systems and the compensation for the low power density of fuel cells [21]. As can be seen in Figure 3, there are two possibilities: serial and parallel configurations of hybrid systems [20].

### 2.4. Mechanical Energy Conversion in UAVs

UAV propulsion systems can be classified by their type of mechanical energy conversion. Since internal combustion is the usual propulsion method, it is separated into jet engines and reciprocating engines according to the fuel used (petrol or diesel).

- Jet engines: they produce thrust and can be classified into jet engines and turbofans. Jet engines (jet-turbine or turbojet, ca. 30 g/(kN·s) [22]) are typical of Unmanned Combat Aerial Vehicles (UCAVs). Turbofans (10–23 g/(kN·s) for small turbofans) [23] are typical in high subsonic and are more used in commercial aviation than in UAVs, notwithstanding, some UAV's such as the Global Hawk HALE UAV use them. If, instead of thrust, power is delivered to a shaft driving a propeller or rotor, they can be classified as turboprop engines (turbopropeller, ca. 0.49 kg/kWh) where a turbine engine is connected to a traditional propeller (e.g., Predator B UAV MALE) and turboshaft engines (ca. 0.30 kg/kWh) [24], typical of rotary wing aircraft. They deliver torque more smoothly and with less vibration than reciprocating engines, but are not suitable for low speed operations due to their high consumption at partial loads.
- Reciprocating engines: there is a great variety attending to the combustion process (spark-ignition engines, compression ignition engines, etc.), cycle (two-stroke and four-stroke engines), intake manifold pressure (engine naturally aspirated and supercharged engines), air or water cooled, and so forth. In this case, the main classification is determined by the type of working cycle and its corresponding fuel, for example, aviation gasoline (piston-avgas, otto cycle, ca. 0.3 kg/kWh) or diesel (piston-diesel, Diesel cycle, ca. 0.24 kg/kWh) [25]. Among its weaknesses are the vibrations that prevent torque from being delivered smoothly. These engines are suitable for small and medium size UAVs and for short range operations, therefore Maximum Takeoff Weight (MTOW) is typically <1,250 kg [9] (nano-, Micro-, Mini-, Close Range (CR), Short Range (SR), Medium Range (MR), and Medium Range Endurance (MRE) UAVs are included in these categories).
- Electric motors: they convert electricity into mechanical energy by moving a propeller, fan, or rotor. Electrical energy is supplied by a battery, photovoltaic or fuel cell. They have the advantage of being the quietest and having one of the lowest thermal signatures. Currently, mainly Micro- and Mini-UAVs are powered by batteries and electric motors [19]. Although they are undergoing continuous improvement, electricity demand comes not only from the engine, but also from the payload and communication systems, limiting the endurance or speed. Fuel cells and photovoltaic cells have already been tested in UAVs, but they remain far from being mature technologies.
- Other types of engines: The Wankel rotary engine, which operates simply and smoothly, is gaining acceptance thanks to their sealing and torsional vibration problems being solved. Currently, Israeli Elbit Hermes 180 and Hermes 450 UAV with 28 and 38 kW, respectively, use these types of engines; they have high durability and a specific consumption of 0.35 kg/kWh [19].

### 2.5. Examples and Trends. Analysis

In order to compare different types of mechanical energy conversion components used in UAVs, in terms of power densities, technical data have been collected and represented in Table 1.

**Figure 4:**
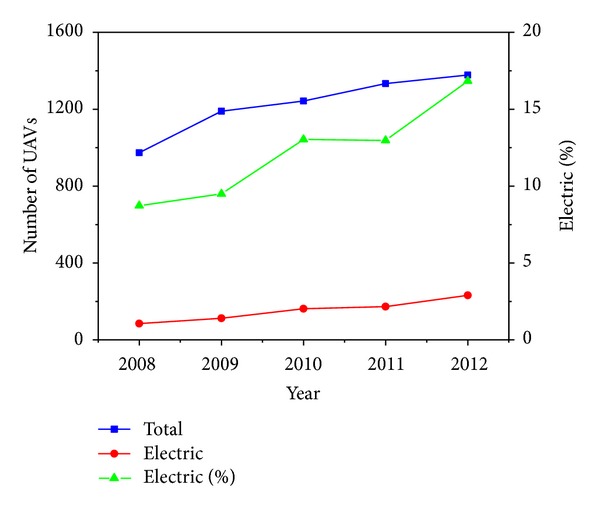
2008–2012 evolution with % electric UAVs trend.

**Figure 5:**
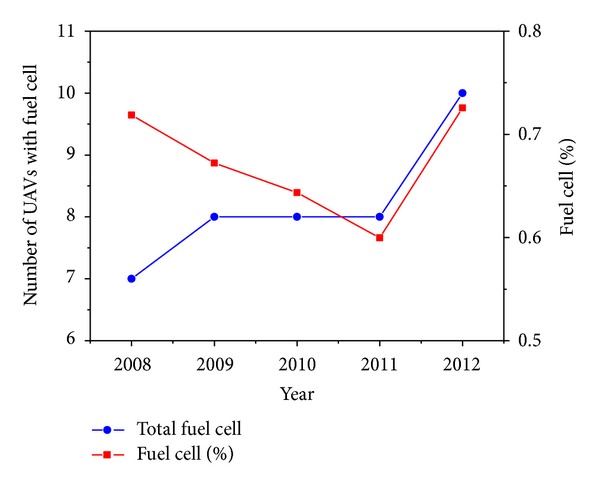
2008–2012 evolution with % fuel cell UAVs trend.

In 2008 there were 85 electric-powered UAVs whilst in 2012, there were around 232 [9] and, as can be seen in Figure 4, these follow a growing trend. “Electric” includes batteries, fuel cells, and solar energy. In Figure 5 the number of fuel cell powered UAVs has been represented. Although quite low, a steady growing trend is observed.

**Figure 6:**
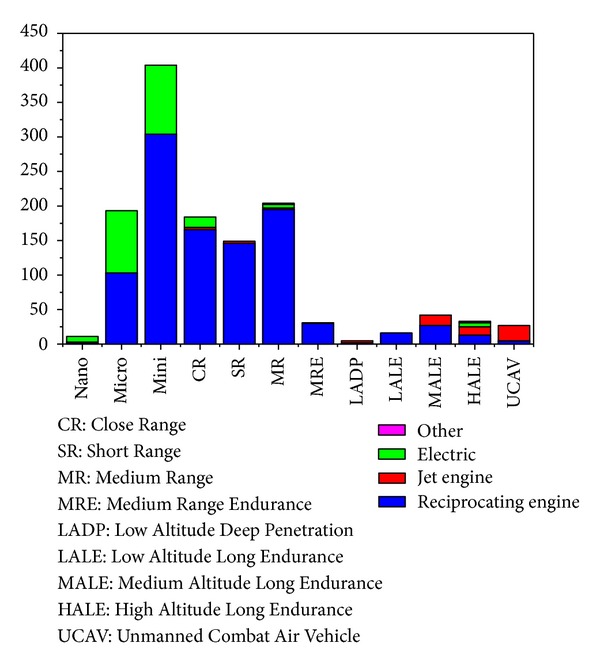
UAVs histogram according to sizes and powerplants. “Electric” includes batteries, fuel cells, and solar. “Other” includes rocket propelled, Wankel, laser powered, and hydrogen combustion.

After observing the evolution in the use of electric motors and fuel cells in UAVs, it is important to check the breakdown (according to [9]) by size and type of propulsion system of existing UAVs in 2012, which is shown in Figure 6. As can be observed, there are many factors to consider when choosing the powerplant for a UAV. With no reference book or guide currently available, it is therefore necessary to make an exhaustive assessment of the requirements in each particular UAV.

In any case, the engine is only one component of the propulsion system. It must be mounted on the UAV and it must be provided with ignition or starting means, fuel supply, its required cooling control, and exhaust gas management if required. All these facts will influence the final choice. When comparing power and energy densities not only must the weight of the engine be taken into account, but also the weight of the energy storage system and the engine's auxiliary systems. In large UAVs, the engine will therefore have a great size and will probably come with its large associated subsystems. However, in a small UAV there will probably be a need for space saving and, consequently, a subsequent selection of those subsystems.

With regard to electric propulsion systems, batteries are currently limited to 150–200 Wh/kg and are expected to show an increase of up to 300 Wh/kg within the next few years; one order of magnitude lower than the specific energy density expected in hydrogen fuel cells [26]. The weight of the electric motor and other auxiliary systems is not included; more information is therefore required in order to properly compare these systems. Unfortunately, since some authors and manufacturers do not give out detailed information about these systems, the weight of storage tanks and auxiliary systems, such as control systems, is generally unknown. In the case of tanks and batteries, for a given propulsion system, manufacturers often usually offer different sizes, thus providing different endurance values.

Recent advances in weight saving of electric motors and batteries have allowed for electric propulsion to be more competitive. The main drawback is the low specific energy density of batteries, resulting in large volumes (around four times the equivalent volume of fossil fuels for a given energy). Thus, the problem relies on the endurance of these vehicles.

The breakthroughs in internal combustion engines are focused on downsizing and HCCI technology (Homogeneous Charge Compression Ignition). This technology is based on producing autoignition of a lean and homogeneous mixture (air/fuel ratio >25) at multiple points in the combustion chamber [26–28].

UAVs are not manned and they typically carry light payloads (e.g., surveillance and communications), so the sum of the propulsion system and the fuel usually exceeds one-third of the total UAV weight, a higher proportion than in conventional aviation [19]. Any slight reduction in the propulsion system weight or in specific consumption can therefore have a significant effect on the endurance increase or the downsizing of the UAV. Current research focuses on the improvement of internal combustion engines and on the use of new energy sources, through fuel cells, for example.

## 3. Fuel Cells in Unmanned Aerial Vehicles Propulsion

### 3.1. Fuel Cell Fundamentals

Among the most interesting alternative propulsion systems for UAVs are those based on fuel cells. It is still an immature but growing technology, with room for improvement in weight, volume, and cost reduction. Fuel cells are electrochemical systems that convert the chemical energy contained in fuels directly into electric energy. When fed with hydrogen, they produce no greenhouse gases, the only products being water and heat, and the level of noise generation by the engine is low. Water, as well as heat and low oxygen-containing exhaust air, is side product of the fuel cell that could have other applications to compensate the weight disadvantages (particularly in large UAVs), such as water supply for other subsystems, deicing, or inerting of a fossil fuel tank [18]. Unlike batteries, a fuel cell does not need to be recharged; it keeps operating while fuel and oxidizer are supplied from the outside. The fuel cell itself consists of an anode where fuel is injected (usually hydrogen, ammonia, or methanol) and a cathode where an oxidant is introduced (usually air or oxygen), separated by an electrolyte ionic conductor [29]. Usually, fuel cells produce low voltages and must be assembled into a fuel cell stack in order to reach the power required for most UAV applications. Since fuel cells are by their nature modular devices, their power can go from microwatts to megawatts, making them useful in a variety of applications.

The main advantages of fuel cells are their low emissions, high efficiency, modularity, reversibility (this property is exploited in RFC Systems) [30], fuel flexibility, range of applications, low noise, and low infrared signature. However, their disadvantages include cost, sensitivity of the electrode catalyst to poisons, their experimental state, and the lack of H_2_ availability. In systems powered by reciprocating and jet engines, fuel only contributes to propulsion in 18–25% of its energy, while in fuel cell powered aircraft this efficiency is in the region of 44% [31].

### 3.2. Types of Fuel Cells and Their Suitability for UAVs

Different types of fuel cells exist, differing in the operational temperature range and the electrolyte used. Combining these types of fuel cells with other elements, new concepts emerge, such as, RFC Systems, where the stack uses its reversibility mode as an electrolyzer or as a fuel cell (e.g., Helios UAV/NASA solar power combined with day/night cycles), or hybrid systems, which rely on batteries when more maneuvering power is required [32]. The different characteristics of each type of fuel cell define what application they are most appropriate for. High temperature fuel cells are usually not considered appropriate for UAVs due to their large size and weight, large start time, and the auxiliary systems required to manage the dissipated heat. Nonetheless, there have been some attempts at miniaturizing UAVs, by using a combination of RFC and solar power, suitable for long endurance flights (up to 5 years, project Boeing/DARPA with Solid Oxide Fuel Cell, SOFC [33]).

In addition to the UAVs, general aviation and commercial transport aircraft are possible applications for fuel cells. Several projects like Antares DLR-H2, developed in 2009 by the German Aerospace Center, or the first flight of a manned fuel cell aircraft performed by Boeing's Madrid branch in 2008 are examples of general aviation applications. On the other hand, A320 ATRA and ENFICA-FC [34] are projects that have explored the substitution of commercial aircraft elements like Ram Air Turbine (RAT) or Auxiliary Power Unit (APU) [35].

SOFCs are high temperature fuel cells (above 800°C). The development state of SOFCs is inferior to that of PEMFCs, apart from being heavier. Therefore this paper will focus on PEMFC. However, reforming and cleaning of kerosene for SOFCs are relatively simple [18]. Therefore SOFCs could have interest for future aircraft applications, since kerosene will still be the most important fuel in the next years.

PEM fuel cells operate at low temperature (typically 50–70°C); the use of polymeric electrolyte provides high current densities. They have a quick start-up, a high specific energy density, and a low specific power density. Therefore, they offer potential advantages for low maneuverability and high endurance UAVs, which is the type of UAV most developed today (mostly used in surveillance applications). The reason is that these properties allow for the development of relatively light, low cost, and reduced volume systems compared to other types of fuel cells [36]. Also, PEM fuel cells have reached a more mature market and several commercial applications already exist [4, 36, 37]. In addition to the aforementioned, the efficiency and environmental benefits, low noise, and thermal signature of these cells are invaluable advantages in the military field. The fuel used is hydrogen and can be stored by different means (compression, liquefaction, metal, or chemical hydrides). Hydrogen is usually obtained from natural gas due to economic reasons, but it is also a renewable fuel as it can be produced from water using solar or wind power. Disadvantages of hydrogen include generation costs (it is an energy carrier, not an energy source), the compression/liquefaction costs of storage due to the extra weight and volume needed, and distribution and safety issues like the risk of explosion. Nonetheless, its main advantage is that it has a high heating value and the reaction product is only water and heat.

Direct Methanol Fuel Cell (DMFC) stacks, a class of PEM fuel cells directly fuelled with methanol, have about half the efficiency (higher heat losses) and power density than PEM fuel cells, but higher energy density (greater endurance) and simplicity [29, 36]. In addition, the storage system is lighter than in the case of hydrogen and the logistics are simpler, potentially able to take advantage of the fossil fuels distribution network. Added to this, the handling and storage of methanol are much more advantageous than that of hydrogen, which requires heavy deposits those must withstand high pressures. Although methanol is toxic, it is not a problem if the appropriate protocols are followed. Another advantage is that it can be obtained from biomass (e.g., wood distillation) and could therefore be considered a renewable fuel [38]. It only emits water and CO_2_, and if distilled, this is reabsorbed by the biomass produced to generate the new methanol, thus closing the cycle. With costs being lower than for hydrogen, there are already DMFC stacks for terrestrial applications and these could be applied in very light low maneuverability UAVs. The market trend for UAVs is precisely towards miniaturization. Given its low power density and in case of specific requirements of high power demand, DMFC (and PEMFC) will probably need to be combined with hybrid battery systems (except in systems with low power demands, such as airships). Of these, lithium-ion batteries are currently the most advanced and developed.

For very small UAVs, compressed hydrogen systems are not practical, since they are not downwardly scalable. In this case, it is preferable to use noncryogenic liquid fuels such as methanol or chemical hydrides, where fuel storage and delivery are considered more critical than the battery performance itself.

**Figure 7:**
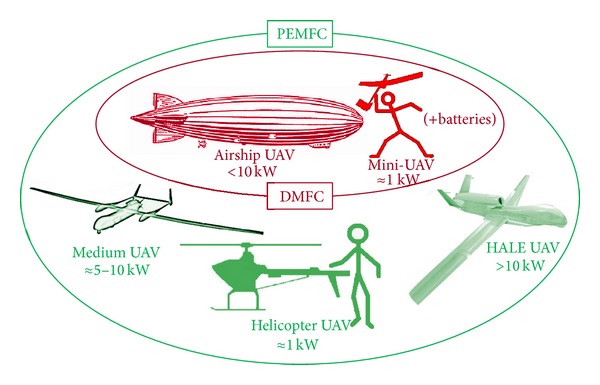
Possibilities of implementing fuel cells propulsion systems in UAVs.

Developments in which a fuel cell delivers power to an electric motor, which in turn drives a propeller or a rotor, already exist, such as a helicopter UAV powered by a PEM fuel cell fed with compressed hydrogen. Recent advances in compressed hydrogen powered PEM fuel cells have achieved power densities of up to 1.4 kW/kg in the 100 kW range, although for the 1 kW range, only 250 W/kg is commercially available [14]. These figures lose around another 20% when the weight of the electric motor is added, leading to final figures worse than other systems. Moreover, the mass of hydrogen, added to its storage system, further worsens the figures with respect to fossil fuels; the data on methanol systems are not so unfavorable regarding the storage system. However, the energy conversion process is very efficient compared to internal combustion engines and great endurance is achieved. This is due to its high energy density, making them very suitable for long endurance surveillance missions. As an example, the Puma UAV equipped with a Protonex PEM stack flew 9 hours, improving on the 2-hour flight of the same battery-electric UAV [39].  Figure 7 summarizes the possibilities of implementing fuel cell propulsion systems in UAVs.

Auxiliary systems of a fuel cell powered UAV include heat management, humidification system, tanks, and controller. The weight of these systems is very variable and can reach from 14.83% (UTRC UAV helicopter demonstrator) [14], 43.25% (Georgia Tech UAV) [40] to 83.33% (Ion Tiger NRL UAV) [41]. Auxiliary systems consume part of the gross power (about one sixth in the Boeing Fuel Cell Demonstrator manned airplane) [42].

Airships are also a potential new application for fuel cells. There is a rebirth of airships resulting in various studies and prototypes, especially in the field of monitoring and surveillance, where the latest technological advances in materials and navigation are being incorporated [43]. The requirements in the propulsion of airships are relatively modest, being logically higher for takeoff and landing. Due to the buoyancy conferred by helium, they are more energy efficient and cheaper to operate than Heavier-Than-Air (HTA) aircrafts when transporting payloads. Traditionally, they have worked with internal combustion engines, but they are best suited for long flight time in all regimes (including reverse). Nonetheless, they usually run at much lower power than nominal, making them inefficient. In addition, piston engines in airships have lubrication problems [44]. Therefore, electric motors offer good prospects and fuel cells could play an important role. Specifically, DMFC would be suitable for airships because of its long-range and low power density. An example is a Lockheed Martin, High Altitude Airship Project that will operate in a geostationary position above the jet stream (an advantage over combustion engines which exhibit a maximum operating altitude), delivering persistent station keeping as a surveillance platform. A 137 m length airship with a RFC solar/H_2_/O_2_ propulsion system would be able to remain 10 years flying and be an alternative to satellites [45]. High Altitude Airship has altitude restriction because the oxidizer is stored onboard. Most fuel cells are however airbreathing to save weight and storage volume and as such also have an altitude restriction.

### 3.3. Advantages and Disadvantages of Fuel Cells in UAVs

Thus, the advantages of a fuel cell powered electric motor can be summed up as follows:

- more efficient than fossil fuel technologies,
- high energy density, which means greater endurance,
- reliability: few moving parts and easy automation,
- flexibility of operation: in that they are reversible, can work at high performance without interruption for a wide range of power demands, and can also rapidly change their power output; anyway, the latest point is not enough for demanding maneuvers like takeoff, thus the support, with batteries is often necessary,
- modular and easy to implement,
- direct energy conversion (no combustion),
- negligible noise and vibration,
- low or zero emissions,
- a variety of applications: in addition to propulsion systems in UAVs, they might be used in APUs, auxiliary power systems, ground control stations, and so forth,
- water, as well as heat and low oxygen-containing exhaust air, is side product of the fuel cell that could have other applications to compensate the weight disadvantages (particularly in large UAVs), such as water supply for other subsystems, deicing, or inerting of a fossil fuel tank.

Main disadvantages are

- high cost: it is not yet a mature technology and uses expensive materials like platinum, used as a catalyst,
- sensitivity to fuel contamination, requiring expensive filtering systems,
- the need for qualified maintenance personnel,
- low power density compared to other systems, especially in DMFC stacks,
- unavailability of hydrogen: H_2_, one of the fuels used in fuel cells, is not naturally abundant; it must be obtained through water electrolysis or hydrocarbons reforming, defining it as an energy carrier rather than an energy source; there is also currently no distribution infrastructure,
- unproven reliability for commercial use: there is little “real” commercial fuel cell UAVs and where they exist, their implementation is very recent, and their technology is therefore still far from mature,
- safety issues regarding H_2_ handling (hydrogen gas forms explosive mixtures with air) and methanol toxicity, this fact has influence, for a military setting, on the logistics of the fuel supply in the battlefield.

With the reasons given above, the advantages and disadvantages of a UAV propulsion system based on fuel cells are practically the same as those of the fuel cell stack itself, slightly worsened by the need to add the electric motor and its accompanying weight.

### 3.4. Examples in the Market.

**Figure 8:**
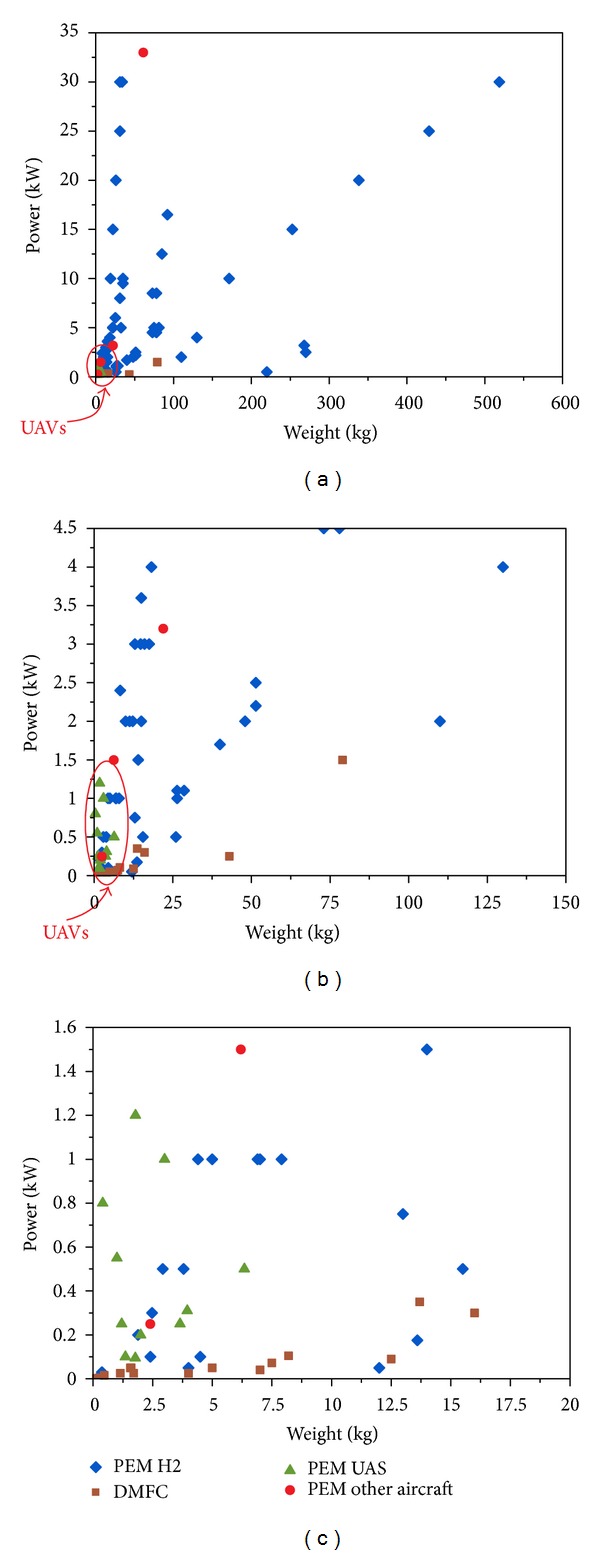
Power versus weight of PEM and DMFC commercial fuel cells.

*Analysis*. In Figure 8, power versus weight of PEM and DMFC commercial fuel cells is represented. Tank weight has been included when available. The cases of PEM fuel cells with UAV and general aircraft applications are considered. It can be seen that there are PEM fuel cells that could easily be adapted to UAVs, but no DMFC for UAV application has yet been identified.

The main problem of fuel cells is their low power density, meaning the UAV performance would have restrictions and would be very dependent on its aerodynamic design, weight, and fuel cell performance [46]. This highlights the possible complementary use of batteries to form hybrid systems, especially in the case of DMFC which have these lower power densities. There are no examples found of a UAV powerplant based on a DMFC stack (not even hybrid), only a few experimental developments in their early stages [47, 48]. There are examples of hydrogen-based PEM UAVs, as already mentioned (10 in 2012 according to UVS [9]), and UAV hybrid projects based on compressed hydrogen PEMs [32]. In Figure 8, the power of commercial fuel cells versus their weight is plotted for three different scales of different UAV application cases. PEMFCs are all located in the left lower corner of Figures 8(a) and 8(b). There is one existing example of a big UAV with a PEM powerplant, the Aerovironment Global Observer, but no technical data were found regarding the powerplant, so it is not shown in figures. Figure 8 also shows that DMFC found in the market have very low power and that, with the current technology, a fuel cell can only be implemented in small UAVs.

Relevant technical data of PEM fuel cells for UAV applications have been collected and summarized and are shown in Table 2. They are important in order to confirm the viability of fuel cells for UAV powerplants and allow the comparison between different types of fuel storage systems related to the sizes and fuel cell power densities of UAVs.

Nowadays, the specific energy density of a PEM fuel cell system, which implies higher endurance in UAVs, is about 700–1000 Wh/kg, but it is to be increased up to 10 kWh/kg within the next 10–15 years and to 20 kWh/kg within 20–30 years, which, if achieved, will enable the all-electric flight of a large commercial aircraft [34]. In the case of DMFC cells, the advantage in terms of endurance comes from the methanol storage. The equivalent energy content of 50 gasoline liters is, taking into account the tank mass, 80 kg of methanol while analogous figure regarding H_2_ (300 bar compressed) is 442 kg [49].

Attention should also be paid to the potential performance loss in fuel cells depending on the environmental conditions of the aircraft flight (i.e., changes in pressure/altitude, temperature [50], vibration, humidity, salinity, radiation, shock, etc.). Most fuel cells are designed to work on land, statically, with relatively stable environmental conditions. However, in flight, these conditions change more or less sharply depending on the UAV service ceiling, its speed, airfield location, and so on. The influence on the performance is relevant, since parameters such as humidity will affect the operation of the fuel cell membrane. There is little literature on the influence of environmental conditions on the performance of a UAV ([21, 31, 50], for general aircraft conditions), although the manufacturing company, Horizon Energy Systems, has published data on its technical specification sheets, (UAV Aeropak fuel cell, 200 W, metal hydride cartridge [51]).

## 4. Conclusions

Could the feasibility to implement a fuel cell in a UAV be asserted? The short answer is yes although, obviously, not for all combinations of fuel cells and UAVs. From the study carried out in this work, the following conclusions can be drawn.

- Fuel cell technology is still immature but improving, with clear room for improvement in weight, volume, and costs reductions. Compared with conventional systems, fuel cells offer higher energy density and lower specific power density.
- Fuel cells offer potential advantages in low maneuverability UAVs, which are currently the most manufactured type (e.g., surveillance applications), and in high endurance operations.
- PEM fuel cells have reached a more mature market. The facts of being of low temperature and having a fast start-up time are features consistent with the requirements of most UAVs. Reforming other fuels (e.g., natural gas, gasoline, etc.) remains an uninteresting option because the weight and volume of the reformer would need to be added. A jet fuel reformer could only be assessed in the case of large UAVs [31].
- There are different fuel storage systems for each type of fuel cell. Therefore, in the case of a UAV, it is essential to minimize the weight of the overall propulsion system (fuel cell/storage system) without forgetting to consider the weight of auxiliary systems, such as thermal control or water management systems.
- Several commercial UAV applications of PEM hydrogen fuel cells already exist. There is even an example of a fuel cell powered UAV rotorcraft (demonstrator, [14]). In most of these cases, the target is small size UAVs. Nonetheless, some fuel cell manufacturers already offer fuel cells designed for a general type of UAV and on the other hand there are UAV manufacturers that have developed a UAV specifically designed to accept a fuel cell based powerplant.
- Compared to hydrogen and despite its toxicity which is not an important problem following the proper protocols, methanol has advantages in terms of its storage systems, especially with logistics, in energy density and cost, and even in safety issues. DMFC fuel cell systems have less power density in comparison to other types of fuel cells, but higher energy density. The possibility of implementing DMFC stacks in UAVs is considered an interesting alternative and left for future work.
- Airship propulsion requirements are relatively modest, being the greatest for takeoff and landing. Electric motors offer good prospects with respect to the currently used internal combustion engines, and consequently fuel cells can play an important role. Specifically, DMFC would be suitable for airships because of its long-range and low power density.
- Since the low power density is the main problem of fuel cells, hybridization with electric batteries would be necessary in most cases.

## Figures and Tables

**Table 1:** Examples of power densities of different UAV powerplant components.

Specifications from manufacturers' websites					
Type	Manufacturer/model	Weight (kg)	Peak power (kW)	Power density (kW/kg)	Application
Reciprocating engine (two-stroke)	Rotax 503 [9]	33.2	37	1.11	INTA SIVA UAV (SR)
Turboprop	Honeywell TPE 331-10 [10]	153	671	4.38	PREDATOR B (MALE)
Turbofan	Pratt & Whitney Canada PW545B [11]	347	18.32 kN	10.86	PREDATOR C (MALE, 741 km/h)
Electric motor	ElectriFly GPMG4805 Brushless DC [12]	1.48	8.4	5.68	Radio-Controlled Aircraft
Lithium ion battery	ANR 26650 Cylindrical [13]	—	—	2.60	Portable high power
H_2_ fuel cell	UTRC Gen 1 [14]	1.78	1.2	0.675	Helicopter mini-UAV
H_2 _fuel cell	Protonex Ion Tiger UAV (NRL) [15]	1	0.550	0.550	Fixed-wing CR UAV
Solar array	Several Manufacturers [16]	—	—	0.06	Spacecraft Applications
Wankel	O.S. Engines 49-PI Type II 4.97 cc [17]	0.333	0.934	2.8	UAV Wankel engine

**Table 2:** Examples of PEM fuel cells for UAV applications.

Specifications from manufacturers' websites and [18]					
Fuel type	Manufacturer/model	FC weight (kg)	FC power (W)	FC power density (W/kg)	Application/remarks
Chemical hydride cartridge	Horizon Energy Systems/AEROPAK	3.5	200	57.14	IAI Bird Eye 650 LE UAV 10 A-21 V nominal. 600 W with LiPo batteries. Cartridge Type I: 446 Wh/kg. Type II: 607 Wh/kg. Also used in Bluebird Boomerang mini-UAV and Elbit Skylark UAV.

Sodium borohydride	Protonex/UAV C-250	1.2	250	208.33	500 W peak power with batteries. Fuel 833 Wh/kg hydrated. Cartridge 1.8 kg, 1.5 l.

Compressed H_2_	Protonex/Spider Lion UAV (NRL)	1.77	95	53.67	Spider Lion Micro-UAV 2005, 3-hour flight

Compressed H_2_	Protonex/Ion Tiger UAV (NRL)	1	550	550	Ion Tiger UAV. 550 W FC (1 kg + 3.6 kg tank 0.5 kg H_2_), 26 h 1 m flight record in 2009. Powerplant total weight (including fuel and cooling) = 6 kg. Specific energy 1300 Wh/kg. 26 h endurance

Compressed H_2_	EnergyOr/EO-310-XLE	3.95	310	78.48	Radiant Coral Technologies demonstrator UAV 1st flight February 25, 2013. Hybrid. The weight includes auxiliary systems

Compressed H_2_	EnergyOr/EO-210-XLE	3.65	250	68.49	The weight includes auxiliary systems

Compressed H_2_	DLR/HyFish UAV	3	1000	333.33	HyFish UAV 2007. 0.5 hour flight

Compressed H_2_	UTRC/Gen1	1.78	1200	674.16	Helicopter UAV (October 11, 2009). FC (675 W/kg). Powerplant (500 W/kg). Minicopter Maxi Joker. 20 m flight

Compressed H_2_	BCS/BCS500	6.35	500	78.74	Georgia Tech University UAV 2006. Powerplant weight 12 kg

Compressed H_2_	Horizon Energy Systems/H-100	1.36	100	73.53	Johannesburg University Piper Cub UAV

Sodium borohydride	Protonex/ProCore VI	0.408	800	1960.78	Aerovironment Puma UAV 2008. Endurance 9 h

Compressed H_2_	Horizon Fuel Cell Technologies	5	650	130	Pterosoar micro-UAV 2008. Oklahoma State and California State Universities. 15.5 h endurance. FC 480 Wh/kg

Liquid H_2_	NASA/Sensor Technology/Aerovironment	—	—	—	Aerovironment Global Observer (GO-1). 65,000 ft alt., 7-day endurance. PL 180 kg

## Acronyms

APU: Auxiliary Power Unit
CR: Close Range
DARPA: Defense Advanced Research Projects Agency
DC: Direct Current
DMFC: Direct Methanol Fuel Cell
ENFICA: Environmentally Friendly Intercity Aircraft
FC: Fuel cell
HALE: High Altitude Long Endurance
HCCI: Homogeneous Charge Compression Ignition
HTA: Heavier-Than-Air
LADP: Low Altitude Deep Penetration
LALE: Low Altitude Long Endurance
MALE: Medium Altitude Long Endurance
MR: Medium Range
MRE: Medium Range Endurance
MTOW: Maximum Takeoff Weight
NASA: National Aeronautics and Space Administration
NRL: Naval Research Laboratory
PEM: Proton Exchange Membrane, Polymer Electrolyte Membrane
PEMFC: Proton Exchange Membrane Fuel Cell, Polymer Electrolyte Membrane Fuel Cell
P/L: Payload
RAT: Ram Air Turbine
RFC: Regenerative Fuel Cell Systems
SOFC: Solid Oxide Fuel Cell
SR: Short Range
STRA: Stratospheric
UAS: Unmanned Aerial System
UAV: Unmanned Aerial Vehicle
UCAV: Unmanned Combat Aerial Vehicle
US: United States
UTRC: United Technologies Research Center
UVS: Unmanned Vehicle Systems.
