# Corrigendum to “Fuel Cells: A Real Option for Unmanned Aerial Vehicles Propulsion”

**DOI:** 10.1155/2015/419786

**Published:** 2015-04-23

**Authors:** Óscar González-Espasandín, Teresa J. Leo, Emilio Navarro-Arévalo

**Affiliations:** ^1^Instituto Nacional de Técnica Aeroespacial “Esteban Terradas” (INTA), Carretera Ajalvir km.4., Torrejón de Ardoz, 28850 Madrid, Spain; ^2^Departamento de Sistemas Oceánicos y Navales, ETSI Navales, Universidad Politécnica de Madrid, Avenida Arco de la Victoria 4, 28040 Madrid, Spain; ^3^Departamento de Motopropulsión y Termofluidodinámica, ETSI Aeronáuticos, Universidad Politécnica de Madrid, Plaza Cardenal Cisneros 3, 28040 Madrid, Spain

In “Fuel Cells: A Real Option for Unmanned Aerial Vehicles Propulsion” some unclarities were detected in several figures shown in Table 2. To clarify the table, for the Horizon Energy Systems/AEROPAK, the weight (3.5 kg) has been replaced only by the FC weight (0.47 kg); therefore, the FC power density changes from 57.14 W/kg to 425.53 W/kg. Also, the FC power of PROTONEX/ProCore VI is changed from 800 W (peak power) to 280 W (output power), which implies FC power density of 686.27 W/kg.

We provide the updated Table 2.

## Figures and Tables

**Table 2:** Examples of PEM fuel cells for UAV applications. Specifications from manufacturers' websites and [18].

Fuel type	Manufacturer/model	FC weight (kg)	FC power (W)	FC power density (W/kg)	Application/remarks
Chemical hydride cartridge	Horizon Energy Systems/AEROPAK	0.470	200	425.53	IAI Bird Eye 650 LE UAV 10 A-21 V nominal 600 W with LiPo batteries; cartridge type I: 446 Wh/kg; type II: 607 Wh/kg Also used in Bluebird Boomerang Mini-UAV and Elbit Skylark UAV

Sodium borohydride	Protonex/UAV C-250	1.2	250	208.33	500 W peak power with batteries Fuel 833 Wh/kg hydrated Cartridge 1.8 kg, 1.5 l

Compressed H_2_	Protonex/Spider Lion UAV (NRL)	1.77	95	53.67	Spider Lion Micro-UAV 2005, 3-hour flight

Compressed H_2_	Protonex/Ion Tiger UAV (NRL)	1	550	550	Ion Tiger UAV; 550 W FC (1 kg + 3.6 kg tank 0.5 kg H_2_), 26 h 1 m flight record in 2009 Powerplant total weight (including fuel and cooling) = 6 kg. Specific energy 1300 Wh/kg; 26 h endurance

Compressed H_2_	EnergyOr/EO-310-XLE	3.95	310	78.48	Radiant Coral Technologies demonstrator UAV 1st flight February 25, 2013 Hybrid; the weight includes auxiliary systems

Compressed H_2_	EnergyOr/EO-210-XLE	3.65	250	68.49	The weight includes auxiliary systems

Compressed H_2_	DLR/HyFish UAV	3	1000	333.33	HyFish UAV 2007, 0.5-hour flight

Compressed H_2_	UTRC/Gen1	1.78	1200	674.16	Helicopter UAV (October 11, 2009) FC (675 W/kg) Powerplant (500 W/kg) Minicopter Maxi Joker; 20 m flight

Compressed H_2_	BCS/BCS500	6.35	500	78.74	Georgia Tech University UAV 2006 Powerplant weight 12 kg

Compressed H_2_	Horizon Energy Systems/H-100	1.36	100	73.53	Johannesburg University Piper Cub UAV

Sodium borohydride	Protonex/ProCore VI	0.408	280	686.27	AeroVironment Puma UAV 2008 Endurance 9 h

Compressed H_2_	Horizon Fuel Cell Technologies	5	650	130	Pterosaur Micro-UAV 2008. Oklahoma State and California State Universities 15.5 h endurance; FC 480 Wh/kg

Liquid H_2_	NASA/Sensor Technology/AeroVironment	—	—	—	AeroVironment Global Observer (GO-1); 65,000 ft alt., 7-day endurance; PL 180 kg

